# Supplementary material for: Purine Nucleotide Alterations in Tumoral Cell Lines Maintained with Physiological Levels of Folic Acid
Source: Int J Mol Sci. 2023 Aug 8;24(16):12573. doi: 10.3390/ijms241612573 (PMC10454412; doi:10.3390/ijms241612573)
Supplement: Supplementary file 1 [file ijms-24-12573-s001.zip › ijms-2502385-supplementary.pdf]

Figure S1

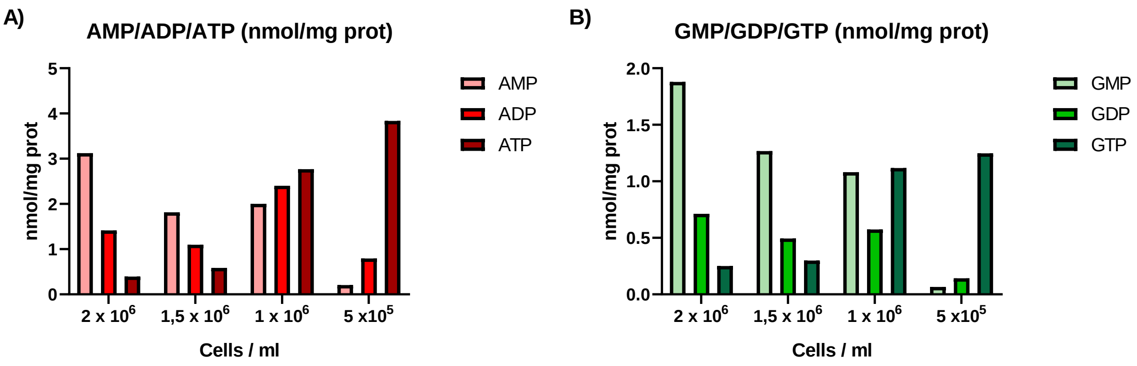

Figure S1. Cellular density has a direct impact in relative concentrations of AMP/ADP/ATP and GMP/GDP/GTP in Jurkat cells. (A) Jurkat cells were incubated for 2 days at different cellular concentrations in RPMI medium containing 2200 nM folic acid (FA). Adenylates levels were determined by HPLC in PCA extracts. Single values represented, expressing the results as nmol/mg protein. **(B)** Jurkat cells were incubated for 2 days as described in (A) and guanylates levels were determined by HPLC in PCA extracts. Single values represented, expressing the results as nmol/mg protein.

Figure S2

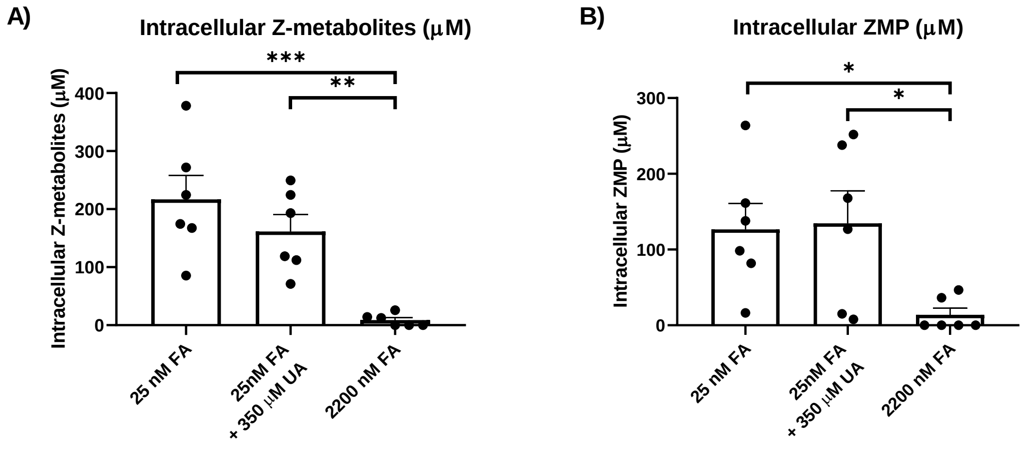

Figure S2. Physiological levels of folic acid induce ZMP accumulation in Jurkat cells. (A) Jurkat cells were incubated for 5 days in RPMI medium containing 25 nM folic acid (FA), 25 nM FA plus 350 µM uric acid (UA), or 2200 nM FA. Cell extracts were obtained with 0.4 N PCA and Z-metabolites determined with the Bratton-Marshall test. The graphs represent the mean ± SEM of 6 independent experiments, expressing the results as µM. Intracellular Z-metabolites concentration was calculated based on the volume of a Jurkat cell. **P < 0.01; ***P < 0.001. One-way ANOVA, uncorrected Fisher's LSD test. (**B)** Jurkat cells were treated with different media, as described in (A), and ZMP levels were determined by HPLC in cell extracts. The graphs represent the mean ± SEM of 6 independent experiments, expressing the results as µM. Intracellular ZMP concentration was calculated based on the volume of a Jurkat cell. *P < 0.05. One-way ANOVA, uncorrected Fisher's LSD test.

Table S1

Table S1: Purine nucleotide levels of Jurkat cells incubated with different media.

Nucleotide \ Condition	25 nM FA	25 nM FA + 350 µM FA	2200 nM FA
	Mean ± SEM	Mean ± SEM	Mean ± SEM
**ATP**	**1742 ± 428***	**1403 ± 143***	**2835 ± 403**
**ADP**	**531 ± 229**	**258 ± 55**	**853 ± 22**
**AMP**	**72 ± 22**	**54 ± 26**	**193 ± 74**
**GTP**	**663 ± 70**	**676 ± 79**	**976 ± 183**
**GDP**	**131 ± 35**	**86 ± 21**	**201 ± 63**
**GMP**	**71 ± 23**	**44 ± 14**	**95 ± 44**
**ZMP**	**127 ± 34***	**135 ± 43***	**14 ± 9**

Table S1. Jurkat cells were incubated for 5 days in RPMI medium containing 25 nM folic acid (FA), 25 nM FA plus 350 µM uric acid (UA), or 2200 nM FA. Cell extracts were obtained with 0.4 N PCA and purine levels determined by HPLC. Results are the mean ± SEM of 6 independent experiments and are expressed as intracellular concentration (µM), based on the volume of a Jurkat cell. ATP>ADP>AMP and GTP>GDP>GMP ratio is conserved in every experimental condition studied. ATP was significantly reduced in 25 nM FA and 25 nM FA plus 350 µM UA compared with 2200 nM FA. ZMP was significantly increased in 25 nM FA and 25 nM FA plus 350 µM UA compared with 2200 nM FA. *P < 0.05. One-way ANOVA, uncorrected Fisher's LSD test.

Figure S3

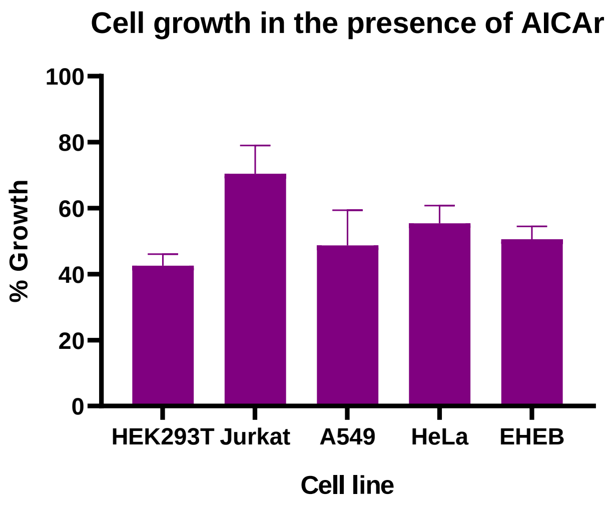

Figure S3. AICAr inhibits cell growth in human cell lines. HEK293T, Jurkat, A549, HeLa and EHEB cells were maintained for 4 days in RPMI containing 2200 nM FA and then incubated with the same medium with or without 2 mM AICAr for 24 h. Cells were counted with a Neubauer chamber. The graph represents the mean ± SEM of 3 independent experiments, expressing the results as % of growth of cells treated with AICAr with respect to untreated cells. One-way ANOVA, uncorrected Fisher's LSD test.

Figure S4

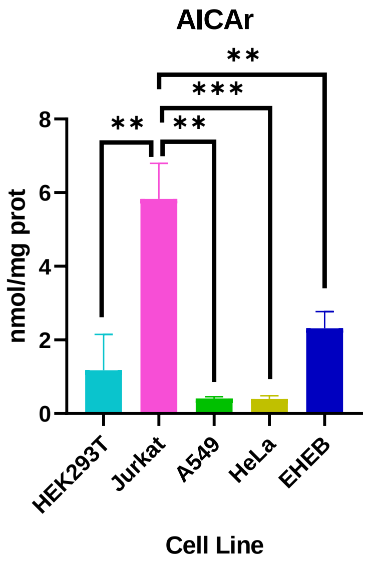

Figure S4. AICAr intracellular concentration in cells cultured with RPMI 2200 nM FA plus 2mM AICAr. HEK293T, Jurkat, A549, HeLa and EHEB cells were incubated for 5 days in RPMI containing 2200 nM FA with 2 mM AICAr for 24 h. Cell extracts were obtained with 0.4 N PCA and nucleotides determined by HPLC. The graph represents the mean ± SEM of at least 3 independent experiments, expressing the results as nmol/mg protein. One-way ANOVA, uncorrected Fisher's LSD test. *P < 0.05; **P < 0.01; ***P < 0.001.

Figure S5

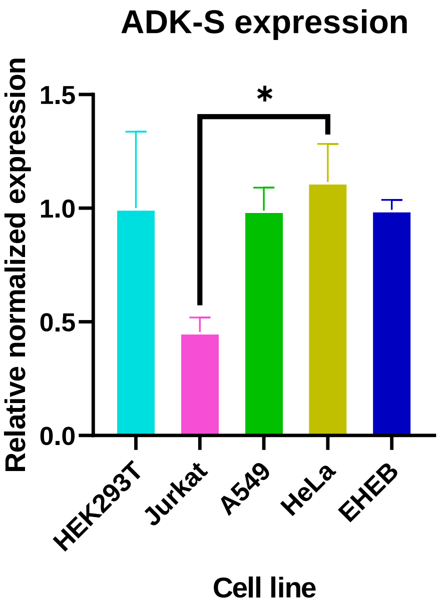

Figure S5: ADK-S mRNA expression in different cell lines. HEK293T, Jurkat, A549, HeLa and EHEB cells were cultured in RPMI containing 2200 nM FA. Total RNA was extracted, and cytosolic adenosine kinase (ADK-S) mRNA levels quantified by qRT-PCR as described in Material and Methods. Results were expressed in arbitrary units (AU), using HEK293T cell line as a reference condition for normalization of the data. The graph represents the mean ± SEM of 3 independent experiments. One-way ANOVA, uncorrected Fisher's LSD test. *P < 0.05.
